# Electrochemical Mechanistic Analysis from Cyclic Voltammograms Based on Deep Learning

**DOI:** 10.1021/acsmeasuresciau.2c00045

**Published:** 2022-08-31

**Authors:** Benjamin B. Hoar, Weitong Zhang, Shuangning Xu, Rana Deeba, Cyrille Costentin, Quanquan Gu, Chong Liu

**Affiliations:** † Department of Chemistry and Biochemistry, University of California Los Angeles, Los Angeles, California 90095, United States; ‡ Department of Computer Science, University of California Los Angeles, Los Angeles, California 90095, United States; § Université Grenoble Alpes, DCM, CNRS, 38000 Grenoble, France; ∥ Université Paris Cité, 75013 Paris, France; ⊥ California NanoSystems Institute, University of California Los Angeles, Los Angeles, California 90095, United States

**Keywords:** electrochemistry, cyclic voltammetry, neural networks, mechanism classification, ResNet, automated analysis, machine learning

## Abstract

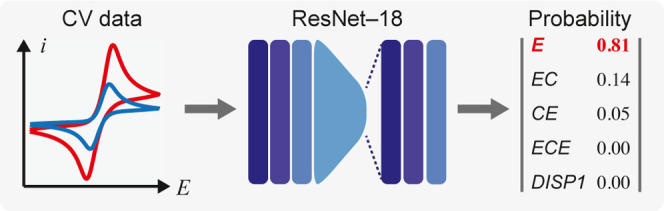

For decades, employing cyclic voltammetry for mechanistic investigation has demanded manual inspection of voltammograms. Here, we report a deep-learning-based algorithm that automatically analyzes cyclic voltammograms and designates a probable electrochemical mechanism among five of the most common ones in homogeneous molecular electrochemistry. The reported algorithm will aid researchers’ mechanistic analyses, utilize otherwise elusive features in voltammograms, and experimentally observe the gradual mechanism transitions encountered in electrochemistry. An automated voltammogram analysis will aid the analysis of complex electrochemical systems and promise autonomous high-throughput research in electrochemistry with minimal human interference.

## Introduction

Cyclic voltammetry is one of the most common electrochemical characterization techniques, and it generates valuable mechanistic information for redox-active chemical systems.^1−3^ For decades, cyclic voltammetry has been indispensable for electrochemical applications in sensing, energy-storage, chemical transformations, and beyond; however, the general protocol of initial mechanistic analysis after experiments has remained largely unchanged since its inception.^3,4^ Researchers manually inspect the shapes and variations of cyclic voltammograms under multiple different scan rates (*v*), sometimes with different reactant concentrations, and subsequently hypothesize a qualitative mechanism consisting of interfacial charge transfers (*E* step) and/or solution reactions (*C* steps),^1,2^ before sometimes extracting quantitative kinetic information via additional experiments and/or numerical simulations.^5,6^ However, such manual inspection demands extensive researcher training, potentially incurs human bias, and is not compatible with automated testing needed for high-throughput screening. An algorithm that automatically analyzes cyclic voltammograms and qualitatively categorizes electrochemical systems into mechanisms with a specific combination of *E* and/or *C* steps will help alleviate the aforementioned challenges in the manual analysis of cyclic voltammograms.

**Figure 1:**
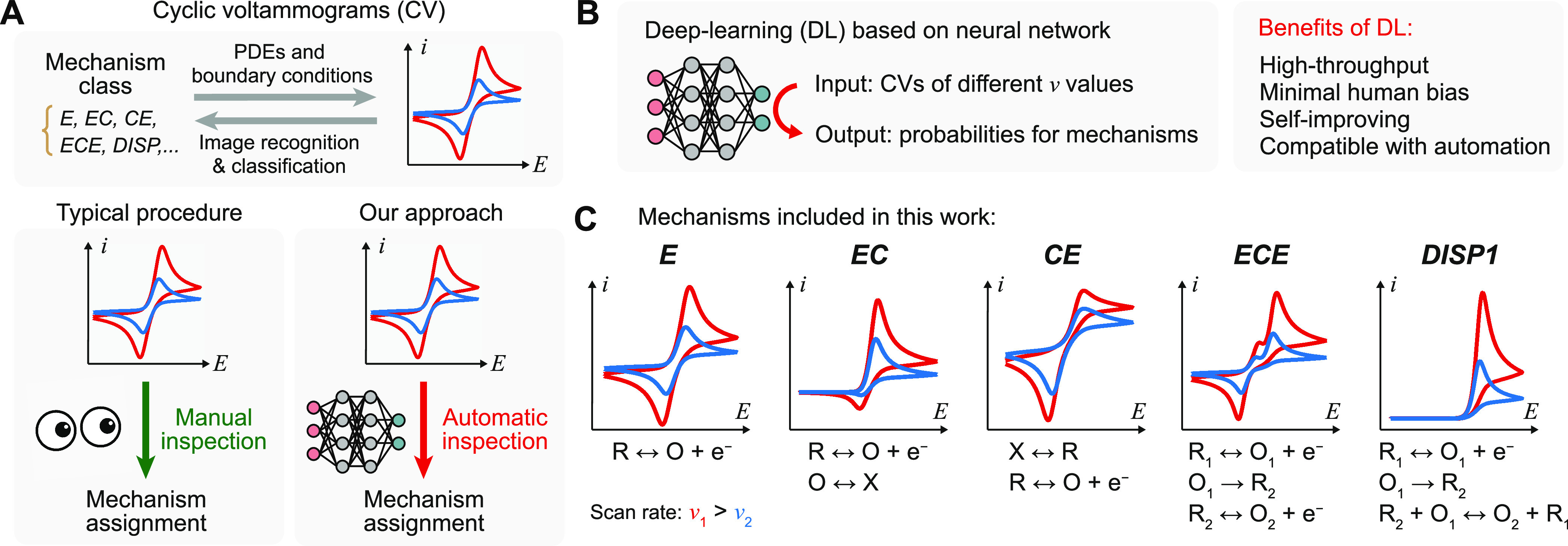
Deep-learning (DL) algorithm for automatically analyzing cyclic voltammetry. (A) Bijective relationship between electrochemical mechanism and cyclic voltammograms, and the comparison between manual inspection of voltammograms and our approach. (B) Function of DL algorithm and its proposed benefits. (C) Five common molecular electrochemical mechanisms included in the DL algorithm. PDEs, partial differential equations.

We envision that machine-learning algorithms such as those of deep-learning (DL) are capable of aiding mechanism categorization in cyclic voltammetry. In electrochemistry, the kinetics of *E* and/or *C* steps formulate the set of partial differential equations (PDE) and boundary conditions that dictate the *i–E* characteristics recorded in the cyclic voltammograms under a collection of different *v* values ({*v*, *i*(*E*)}_*n*_, *n*, number of different *v* values) (Figure 1A).^1,2,7^ Such a mathematically bijective function between electrochemical mechanisms ({*E_i_*, *C_j_*}) and the electrochemically accessible parameter space of the combined voltammograms {*v*, *i*(*E*)}_*n*_ suggests that it is feasible to employ DL algorithms to designate discrete mechanisms from sufficiently sampled cyclic voltammograms with minimal ambiguity (Figure 1A). Indeed, this bijective relationship enables numerical simulations based on finite-element methods,^7,8^ and recently by artificial neural networks,^9−11^ to be used as a tool to efficiently search the parameter space of cyclic voltammograms and fit kinetic parameters upon a mechanism determined a priori by manual inspections of voltammogram. Yet, electrochemical mechanistic investigations remain commonly trial-and-error because the determination of the aforementioned a priori mechanism before any quantitative studies still relies on manual inspection.

We posit that a set of cyclic voltammograms simulated from finite-element methods based on preset mechanism designations will suffice in the first-order approximation for the establishment of a DL model that analyzes cyclic voltammograms and qualitatively categorizes mechanisms provided a large enough sampling of {*v*, *i*(*E*)}_*n*_. Research by Bond and co-workers tested the concept of DL-based automatic analysis of a single simulated voltammogram for a selection of three mechanism types with overall accuracies just below 90%.^12,13^ The exploration of this concept with experimental data, which commonly includes multiple voltammograms at different *v* values, has yet to be conducted for mechanistic studies. We advocate the broader use of DL-based analysis and hypothesize that the bijective relationship between {*E_i_*, *C_j_*} and {*v*, *i*(*E*)}_*n*_ enables the establishment of DL algorithms that detect and utilize subtle voltammogram features, ones not commonly used as mechanistic discriminants by humans, and observe the evolution of “edge” cases when two mechanisms coexist and/or one mechanism is transitioning into another one, challenging scenarios for manual analysis in cyclic voltammetry. The established algorithms can be continuously refined and improved from experimental data, potentially including data contributed by the electrochemistry community. The algorithms will find their use in analyzing complex mechanistic scenarios and addressing the current paucity of automatic, high-throughput mechanistic analysis in electrochemistry.

In this work, we demonstrate a DL algorithm for automatic mechanistic analysis for cyclic voltammetry (Figure 1B). State-of-the-art DL algorithms using residual neural networks (ResNet) architecture^14^ are established to analyze cyclic voltammograms at different scan rates {*v*, *i*(*E*)} and yield the electrochemical system’s probability toward five of the most common stoichiometric homogeneous mechanisms in electrochemistry textbooks (Figure 1C):^1,2,15,16^ a single-electron transfer with any level of reversibility (*E*), an *E* step followed by a *C* step with any level of reversibility (*EC*), an *E* step preceded by a *C* step (*CE*), a system of two *E* steps connected by an irreversible rate-limiting *C_i_* step with the second *E* step being more thermodynamically facile than the first one (*ECE*), and a two-electron transfer that is similar to *ECE* yet the second *E* step is replaced by a solution disproportionation reaction (DISP1). We demonstrate DL’s capability of accurately designating mechanisms in simulated and experimental scenarios, unveiling potential new features in the voltammograms elusive to manual inspection, as well as semiquantitatively observing the gradual transitions of electrochemical mechanisms. The developed algorithm will be applicable to analyze complex electrochemical systems when competing mechanisms are intertwined together. In conjunction with robotic experimentation,^17,18^ the demonstration of automatic mechanistic analysis in cyclic voltammetry presents the possibility of automated high-throughput research to investigate mechanisms in electrochemical systems with minimal human intervention.

## Results

**Figure 2:**
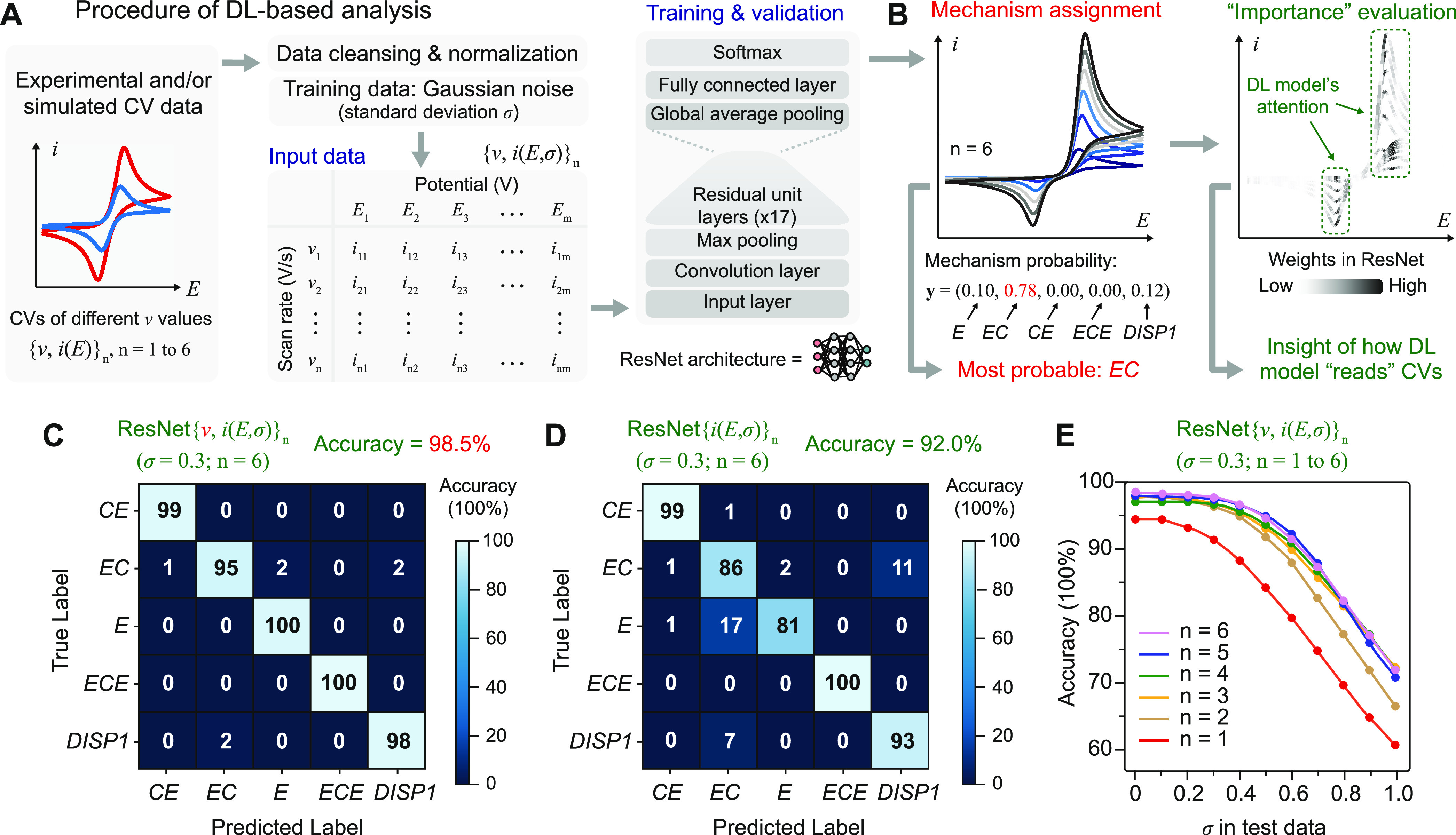
Established DL algorithm of ResNet architecture for cyclic voltammetry. (A) Structure of input data and the convolutional neural networks of residual neural network (ResNet) architecture. (B) Output and utility of DL model for mechanism designation. (C and D) Confusion matrix of DL model trained by simulated cyclic voltammograms (C) with and (D) without explicit values of scan rate (*v*) as input data. *v*, scan rate; σ, the standard deviation of the Gaussian noise; *n*, the number of *v* values. (E) Accuracies of the DL model in panel (C) when tested with simulated voltammograms with varying values of *n* and σ.

The data of cyclic voltammograms were sanitized and transformed into two-dimensional matrices suitable for DL algorithms of ResNet architecture. While cyclic voltammograms are typically presented as images in the literature, much of the white space in voltammograms contains little (if any) information. Hence, similar to the case of electrocardiogram,^19^ a two-dimensional matrix of {*v*, *i*(*E*)}_*n*_, rather than the images of cyclic voltammograms as in the works of Bond and co-workers,^12,13^ is employed to store electrochemical information for the DL-based analysis (Figure 2A). For the ease of training a ResNet, in each set of {*v*, *i*(*E*)}_*n*_, the current densities *i* in voltammograms were normalized as *i*_normalized_ against the largest *i* among all voltammograms in {*v*, *i*(*E*)}_*n*_, with *i*_normalized_ in the forward scan designated as positive values. The electrochemical potentials *E* were adjusted so that the position of 0 V of the adjusted electrochemical potential (*E*_adjusted_) roughly corresponds to the potential of the studied redox couple (see the Methods section). Such data processing ensures a generally readable format of cyclic voltammograms despite the large variations in experimental testing conditions.

Cyclic voltammograms based on the targeted mechanisms (Figure 1C) were numerically simulated as the training set for deep neural networks via the finite-element method (see the Methods section). Numerical models of PDEs, boundary conditions, and initial conditions were constructed based on mechanisms’ definitions in textbooks (see the Supporting Information).^1,2^ Moreover, the numerical models’ parameters, including but not limited to, the numbers and values of scan rate *v* (*n* = 1–6), electrodes’ double layer capacitance reported in the literature,^20^ standard rate constant of interfacial charge transfer in the concentration-dependent Butler–Volmer equation following Nicholson’s formalism (ψ ∈ [10, 0.3]) in the *E* step,^3^ and the equilibrium constants and forward/backward rate constants in the *C* step based on Savéant’s definitions^2^ were incorporated into the simulations and carefully constrained with practical and fundamental considerations. The parameters were randomly sampled (see Table S1 and the Supporting Information) for a comprehensive yet even exploration of the mechanism’s corresponding domain in the {*v*, *i*(*E*)}_*n*_ space, i.e., the corresponding kinetic zone diagrams.^2^ As the experimental voltammograms typically contain Gaussian-type noise due to background and instrumentation,^21^ Gaussian noise of varying degrees of standard deviation σ relative to the maximal current densities were added to the simulated voltammograms, resulting in the training set {*v*, *i*(*E*, σ)}_*n*_ (*n* = 1–6) (examples in Figure S1). The addition of Gaussian noise not only better reflects the realistic electrochemical data but also increases the algorithm’s tolerance toward noises in automatic mechanism categorization^14^ (vide infra).

We chose ResNet,^14^ a widely used network architecture evolved from convolutional neural networks,^22,23^ to extract intrinsic features from high-dimensional data. The ResNet architecture utilizes skip connections within its convolutional layers for deeper networks for greater feature extraction. Such architecture is critical for training successes as it alleviates the problems of both vanishing and exploding gradients during the training process, which mitigates the risk of training failure while maintaining the low overfitting risk inherent in convolutional architectures.^14^ The neural network is trained to take either the first or the second cycle of voltammograms for the same electrochemical system at various numbers of different scan rates ({*v*, *i*(*E*, σ)}_*n*_, *n* = 1–6) and yields the vector ***y*** = {*y*_1_, *y*_2_, *y*_3_, *y*_4_, *y*_5_} (Figure 2B, see the Methods section), in which each component in ***y*** is a surrogate of the electrochemical system’s probability or fraction toward mechanisms of *E*, *EC*, *CE*, *ECE*, and DISP1, respectively. The classification process is completed by designating the mechanism of the largest component in ***y*** as the most probable or most prominent one for the studied electrochemical system. Furthermore, the proposed model can estimate the “importance” of different parts of the voltammograms to the prediction (Figure 2B) by visualizing the relative magnitudes of gradients of the logits on input data after feeding through the neural network. We hypothesize that such a visual guide of algorithm’s “importance” will illustrate how DL models analyze cyclic voltammograms and offer a comparative study between manual inspection and the ResNet-based one.

State-of-the-art ResNet models of 18 residual learning layers, i.e., ResNet-18, were trained and validated by simulated cyclic voltammograms {*v*, *i*(*E*, σ)}_*n*_. Here, the use of a minimal ResNet-18 model is commensurate with our study when smaller neural networks are desired.^14^ When *n* = 6 and σ = 0.3, satisfactory accuracy (>90%) was achieved among {*v*, *i*(*E*, σ)}_*n*_ when more than 3000 electrochemical systems were included for each mechanism type in the training set (Figure S2a). After 1000 epochs of training to improve accuracy (Figure S2b), a voting process that contains eight ResNet-18 models, designated as ResNet{*v*, *i*(*E*, σ)}_*n*_, achieved an overall accuracy of 98.5% for {*v*, *i*(*E*, σ)}_*n*_ (*n* = 6, σ = 0.3) and generated a confusion matrix with nearly zero off-diagonal components (Figure 2C). In comparison, alternative machine-learning algorithms^24^ including linear classification, the vanilla multilayer perceptron (MLP), the MLP using attention mechanism to aggregate the extracted features of each curve (“MLP & attention mechanism”), and the MLP sharing same parameters/weights on the first layer (“MLP & parameter sharing”) only yielded lower accuracies of 88.7, 89.6, 92.3, and 90.7%, respectively (Figure S2c–f). We also built a DL model under the same protocol without the *v* values as input, ResNet{*i*(*E*, σ)}_*n*_ (*n* = 6, σ = 0.3), in which the model’s inputs contain the *n* number of voltammograms but not the exact *v* values. Only an overall accuracy of 92.0% was achieved, and the corresponding confusion matrix contains noticeable nonzero off-diagonal entries (Figure 2D). Consistent with manual inspection, the exact values of *v* are critical in DL to fully differentiate electrochemical mechanisms.

The established DL model is remarkably resilient to appreciable degrees of noise in the simulated cyclic voltammograms. The prediction accuracy of the DL model trained by {*v*, *i*(*E*, σ)}_*n*_ (*n* = 6, σ = 0.3) was tested by simulated cyclic voltammograms (*n* = 6) with varying values of σ ranging from 0.0 to 1.0. The overall accuracy remains mostly constant and higher than 95% until σ ≥ 0.5 (purple trace in Figure 2E). Even at σ = 1.0, when the simulated voltammograms are barely recognizable by manual inspection (Figure S1), an overall accuracy of more than 70% was achieved. Such a tolerance toward noises in cyclic voltammograms is remarkable in comparison to the DL models trained when σ = 0.0 (no noise) and 0.1, since in the latter two models (trained with σ = 0.0 and 0.1), the overall accuracies gradually drop below 40–80%, respectively, at σ = 0.5 of the testing data (Figure S2g,h). Such a gradual decline in accuracy with added noise is indicative of a robust and well-fit model since an overfit model would be expected to perform poorly when added noise in the testing set deviates from the added noise level that it was trained on. The addition of Gaussian noise in model training increases the robustness and sensitivity of the established DL model against data noise that may not be tolerable by manual analysis.^14^

We also evaluated how the value of *n*, i.e., the number of cyclic voltammograms, affects the overall accuracy of DL models of ResNet architecture. The accuracies of DL models trained by {*v*, *i*(*E*, σ)}_*n*_ (*n* = 1–6, σ = 0.3) were tested by simulated voltammograms (*n* = 1–6, respectively) of σ values ranging from 0.0 to 1.0 (Figure 2E). Interestingly, when σ < 0.5 in the testing data, there are no distinguishable differences in overall accuracies when *n* = 2–6, while the accuracies are noticeably lower when *n* = 1 (Figure 2E). Consistent with the diagnostic value of voltammograms’ evolution across different zones in the kinetic zone diagram,^2^ cyclic voltammograms of at least two different *v* values are sufficient for the DL algorithm to accurately designate the reaction mechanism within the parameter space defined in the training set.

**Figure 3:**
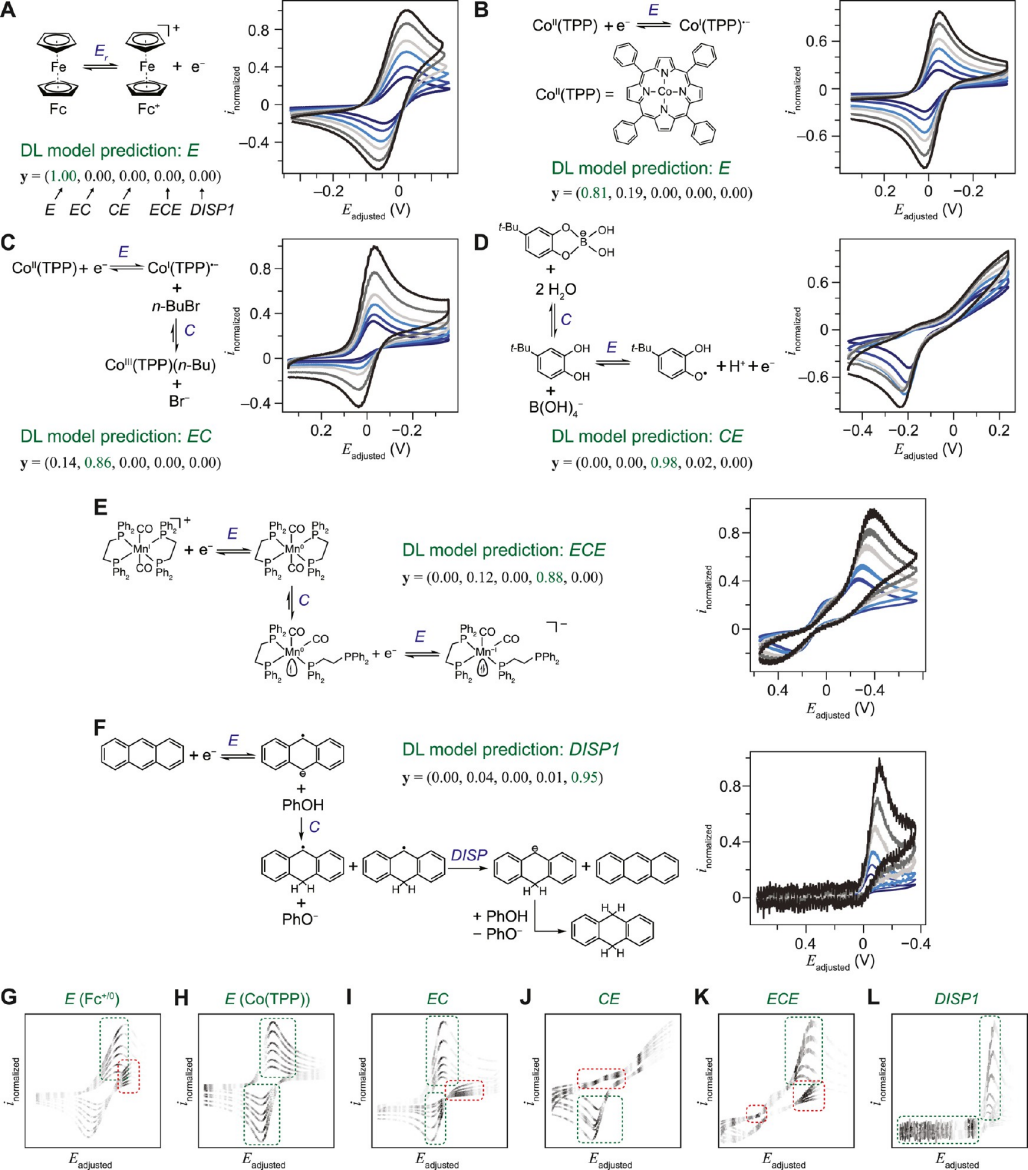
Application of DL model to experimental scenarios. The mechanisms, cyclic voltammograms, ***y*** vectors from DL model’s predictions, and the “importance” plots for (A, G) 1 mM ferrocene/ferrocenium redox couple, (B, H) 1 mM tetraphenylporphyrin cobalt(II) (Co^II^(TPP)), (C, I) 1 mM Co^II^(TPP) and 50 mM 1-bromobutane (*n-*BuBr), (D, J) 1 mM 4-*tert*-butylcatechol in 10 mM pH 9.2 boric acid buffer, (E, K) 5 mM *trans*-Mn(CO)_2_(η^2^-DPPE)_2_^+^ (DPPE, 1,2-bis(diphenylphosphino)ethane), and (F, L) 1 mM anthracene and 0.1 M phenol. *i*_normalized_, normalized current density with the forward scan in the positive direction. *E*_adjusted_, electrochemical potential shifted to center the redox features. The “importance” toward the DL model in expected (green) and somewhat unexpected (red) parts in the voltammograms are highlighted. 3 mm glassy carbon; Ag^+^/Ag reference except panel (D) (Ag/AgCl, 3 M KCl); Pt wire counter electrode. DMF in Ar except panels (D) (water) and (E) (THF). 0.1 M *n*-Bu_4_NClO_4_ in panels (A and E), 0.1 M *n*-Bu_4_NPF_6_ in panels (B, C, and E), and 90 mM KCl in panel (D). The voltammograms of the second (A to E) or the first cycles (F) are displayed and analyzed. *iR* corrected. *v* = 0.05, 0.1, 0.2, 0.3, 0.5, and 0.7 V/s in panels (A, B, C, and D); *v* = 0.1, 0.2, 0.3, 0.5, and 0.7 V/s in panel (E); *v* = 0.05, 0.1, 0.2, 0.5, 1, and 2 V/s in panel (F). Darker traces in panels (G–L) indicate higher “importance” in the DL model.

Encouraged by the ResNet-based model’s accuracy of simulated cyclic voltammograms, we applied the established DL models trained by {*v*, *i*(*E*, σ)}_*n*_ (*n* = 1–6, σ = 0.3) to exemplary experimental scenarios mostly based on the voltammograms of the second cycle. ResNet-based DL model is capable of accurately predicting the *E* mechanism in the ferrocene/ferrocenium (Fc/Fc^+^, 1 mM) redox couple in dimethylformamide (DMF)^25^ (Figure 3A) and the single-electron cobalt(II/I) redox couple with tetraphenylporphyrin cobalt(II) (Co^II^(TPP), 1 mM) as the starting compound in the absence of any electrophiles in DMF^26−28^ (Figure 3B). The DL model accurately recognizes an *EC* mechanism, or more precisely, the *E*_r_*C_i_* variant, when both the thermodynamic and kinetic propensity of the forward *C* step is large enough to be considered an irreversible *C_i_* step, where the addition of 1-bromobutane (*n-*BuBr, 50 mM) as an electrophile to 1 mM Co^II^(TPP) in DMF leads to the formation of tetraphenylporphyrin cobalt(III) *n-*butyl (Co^III^(TPP)(*n*-Bu)) (Figure 3C).^28,29^ The model also accurately recognizes a *CE* mechanism for the first single-electron oxidation of 4-*tert*-butylcatechol (1 mM) in the aqueous buffer of boric acid (pH = 9.2 and 10 mM), where a reversible *C* step is needed to dissociate the thermodynamically favored catechol-borate adduct into the electrochemically accessible catechol^30,31^ (Figure 3D). The ***y*** vector output from the DL model designates the *ECE* mechanism as the most probable one in the case of 5 mM *trans*-Mn(CO)_2_(η^2^-DPPE)_2_^+^ (DPPE, 1,2-bis(diphenylphosphino)ethane) in tetrahydrofuran (THF). This designation is consistent with prior determinations of an *ECE* process with minimal *DISP* contribution,^32,33^ where an intramolecular ligand rearrangement exists between the two single-electron reductions in which the second reduction is thermodynamically more favored than the first one (Figure 3E). Last, from the first-cycle voltammograms, the DL model accurately designates the DISP1 mechanism in the net two-electron reduction of 1 mM anthracene in DMF with the presence of 0.1 M phenol as a proton donor, where the protonation of the anion radical (*C* step) after the first one-electron reductive *E* step is the rate-determining step followed by the disproportionation reaction (*DISP* step)^15,34^ (Figure 3F). The successful mechanism designation by the DL models for model experimental systems suggests the practicality of utilizing DL for automatic analysis in cyclic voltammetry.

## Discussion

There are appreciable similarities between the analytic processes of the established DL algorithm and human inspection. The accuracy decreases from 98.5 to 92.0% when the *v* values were not included as input in the DL model (Figure 2C,D) consistent with manual inspection when a more definitive mechanism assignment is feasible when the explicit *v* values are included in the analysis.^1−3^ Indeed, the exclusion of *v* values as model input deteriorates the model’s accuracies mostly by misassigning *E* as *EC* and misassigning *EC* as DISP1 (increasing from 0% chance to 17% and from 2 to 11%, respectively, from Figure 2C,D). These misassignments are common when *v* information is missing in manual analysis, owing to the gradual transition of cyclic voltammograms between *E* and *EC* in the kinetic zone diagrams as well as the similarity between the one-electron *EC_i_* and two-electron DISP1 processes.^2^ The ResNet-based model’s dependence of prediction accuracies on *n* (Figure 2E) is understandable yet informative. It is common in qualitative mechanistic studies to obtain cyclic voltammograms under multiple *v* values (i.e., *n* > 1) and compare the voltammograms’ evolution.^4^ Yet, in practice, the number of *v* values needed for mechanism determination seems ill-defined. What the DL model suggests is that statistically, in most scenarios, two cyclic voltammograms of different *v* values will suffice and there are diminishing returns of prediction accuracy when *n* ≥ 2 within the parameter space defined in our training set of simulated voltammograms. When *n* = 2, we derived the corresponding mathematical requirements for the two *v* values to be satisfied in the training data set, hence empirically offering good accuracy of mechanistic prediction from our DL model (see the Supporting Information). Such mathematical relationships will be helpful for researchers when deciding on experimental parameters in cyclic voltammetry.

Plotting the algorithm’s “importance” toward parts of cyclic voltammograms suggests that there is additional information in subtle voltammogram features that may elude manual inspection. Figure 3G–L plots the “importance” distributions in cyclic voltammograms from the DL model shown in Figure 3A,F, respectively. Additional “importance” plots of exemplary simulated voltammograms are available in Figure S3. While an understandable amount of the model’s attention is attributed to the presence or absence of primary redox peaks in voltammograms (green areas in Figures 3G–L and S3), one noticeable feature unique to the DL algorithm emerges. An appreciable amount of “importance” of the DL model is frequently assigned to the reverse scan roughly beneath the redox peaks (red areas in Figures 3G–L and S3), an area typically not carefully examined in the manual inspection. We propose that such seldomly examined regions in cyclic voltammograms contain useful mechanistic information and ought to be better utilized for mechanistic studies, probably by DL-based automatic analysis, thanks to the algorithm’s sensitivity. Additional investigations will be conducted to examine such an argument with a more rigorous and systematic analysis.

**Figure 4:**
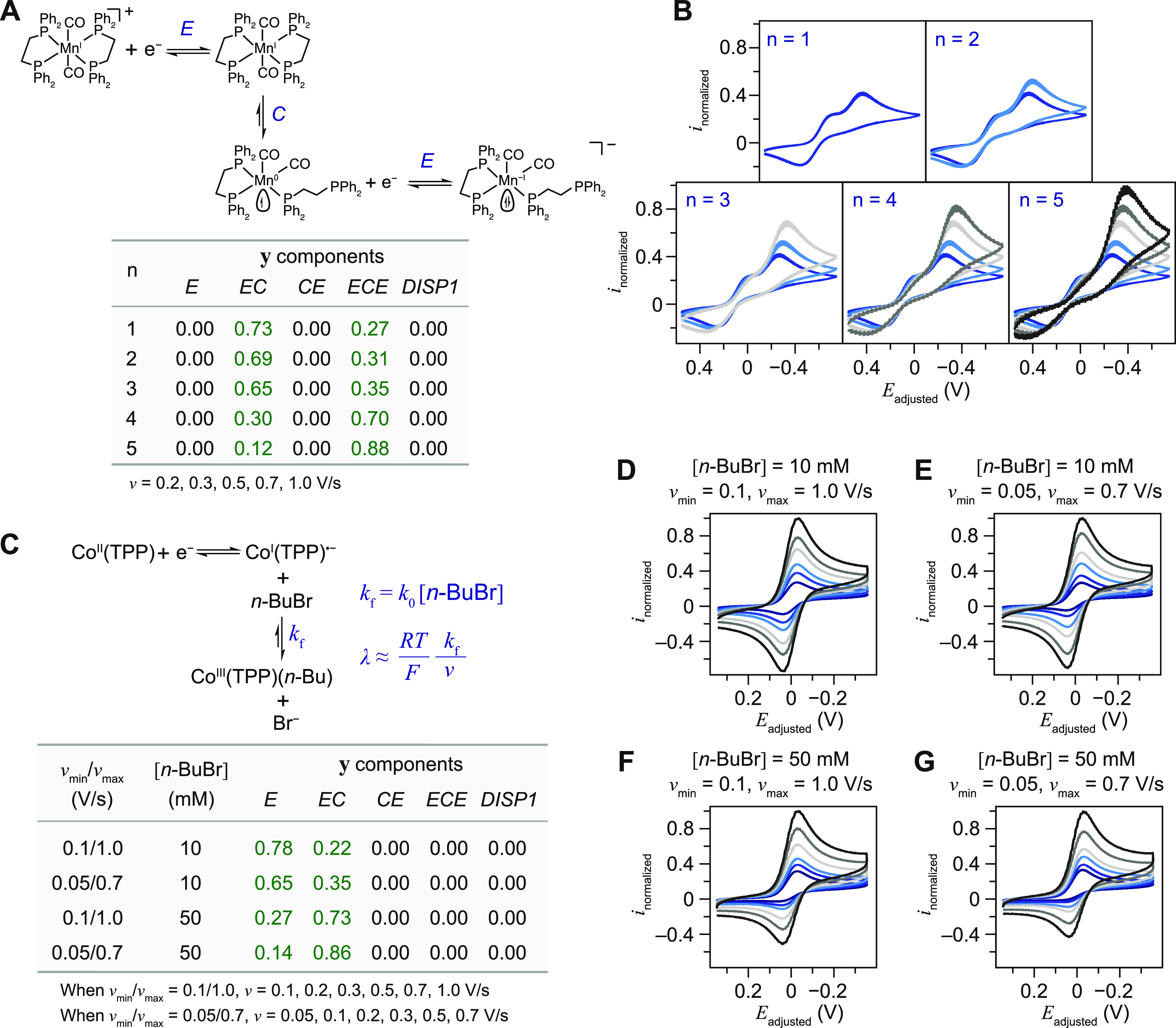
Semiqualitative analysis of cyclic voltammograms with DL algorithm. (A) Mechanism and ***y*** vector components for the *ECE* scenario of *trans*-Mn(CO)_2_(η^2^-DPPE)_2_^+^. (B) Cyclic voltammograms of 5 mM *trans*-Mn(CO)_2_(η^2^-DPPE)_2_^+^ for different *n* values under the same conditions in Figure 3F. (C) Mechanism, kinetic factors, and ***y*** vector components for the *EC* scenario of Co^II^(TPP) and *n-*BuBr. (D–G) Cyclic voltammograms of 1 mM Co^II^(TPP) under different *v* ranges and *n-*BuBr concentrations ([*n-*BuBr]) under the same conditions in Figure 3C. The voltammograms of the second cycle are displayed and analyzed.

In the predominantly *ECE* case of *trans*-Mn(CO)_2_(η^2^-DPPE)_2_^+^ in THF,^32,33^ the evolution of ***y*** vector as *n* increases illustrates how more definitive mechanism determination in edge cases will benefit from cyclic voltammograms of multiple *v* values (Figure 4A,B). With additional voltammograms, the *E*_r_*C*_r_ component in the yielded ***y*** vector decreases from 0.73 (*n* = 1) to 0.12 (*n* = 5) while the *ECE* one increases from 0.27 (*n* = 1) to 0.88 (*n* = 5) (Figure 4B). Such changes in ***y*** components as a function of *n* are consistent with the reported challenges in differentiating *E*_r_*C*_r_ and *ECE*/DISP1 mechanisms with a small number of *v*.^2^

The output ***y*** vector from the DL model can be utilized to provide additional semiquantitative analysis in addition to its function of designating the most probable mechanism. We propose that nonzero probabilities/fractions for mechanisms other than the most probable/prominent one suggest either a competing reaction or a gradual transition from one mechanism to another. In the one-electron reduction of Co^II^(TPP) in the presence of *n*-BuBr (Figure 4C), the forward *C* step, namely the nucleophilic attack of Co^I^(TPP)^•–^ toward *n*-BuBr, is pseudo-first-order on the concentration of *n*-BuBr ([*n-*BuBr]) and the forward rate constant *k*_f_ is proportional to [*n-*BuBr].^28,29^ The corresponding dimensionless kinetic parameter λ, proportional to *k*_f_/*v* (Figure 4C), measures the competition between the solution reaction and redox specie’s diffusion from/to the electrode surface.^2^ As [*n-*BuBr] increases or *v* decreases, λ is larger and leads to more pronounced “irreversibility” from the *C* step in the voltammograms. Experimentally, we found that the cyclic voltammograms display a smaller tendency of reoxidation of Co^I^(TPP)^•–^ when [*n-*BuBr] increases from 10 mM (Figure 4D,E) to 50 mM (Figure 4F,G) and when the range of *v* values decreases from *v* ∈ [0.1, 1.0] V/s (Figure 4D,F) to *v* ∈ [0.05, 0.7] V/s (Figure 4E,G). Accordingly, as shown in Figure 4C, the *EC* component in the yielded ***y*** vectors gradually increased from 0.22 for Figure 4D to 0.86 for Figure 4F, while the component for *E*_r_ correspondingly decreased from 0.78 to 0.14. Such results indicate that the DL model is capable of semiquantitatively detecting the extent and gradual increase of the *C* step, which could be valuable when studying systems with undesirable deactivation in redox cycling or desirable chemical transformation amid an *E*_r_ system.

We speculate that the DL algorithm based on a set of voltammograms is capable of addressing, at least partly, the mechanistic ambiguity noted as “heterogeneous equivalent” by Feldberg and co-workers,^35^ in which different reaction mechanisms may yield similar voltammograms that are indistinguishable within measurement errors. While the issue of “heterogeneous equivalent” is most prominent under a single voltammogram due to the limited information available from electrochemical measurement, our DL algorithm is based on a bijective relationship between mechanism and a more informative-rich set of voltammograms under different scan rates ({*v*, *i*(*E*)}_*n*_). Hence, we contend that the issue of mechanistic ambiguity will be alleviated if not mitigated in the DL algorithm, which will be further evaluated in future studies. Yet, the *EC* case of the one-electron reduction of Co^II^(TPP) in the presence of *n*-BuBr is already sufficiently exemplary (Figure 4C–G). In Figure 4D, the voltammogram at the largest scan rate *v* (1.0 V/s, black trace) can be seemingly interpreted as a quasi-reversible *E* process, while mechanistic ambiguity is resolved by the data at smaller *v* values. Meanwhile, the DL algorithm assigns 0.78 and 0.22 for *E* and *EC* processes, acknowledging the possible existence of mechanistic ambiguity yet offering statistically meaningful diagnostic results useful for researchers. Such a probability-driven approach avoids the pitfalls of deterministic mechanistic assignment and will be beneficial toward addressing the issue of mechanistic ambiguity in the long run.

## Conclusions

Electrochemical analysis for mechanistic investigation has relied heavily on manual inspection, which demands extensive training and may be prone to human bias and misinterpretation due to researchers’ prior experience. In this work, we demonstrated a DL algorithm based on ResNet architecture that automatically analyzes cyclic voltammograms and, congruent with manual inspection, suggests the most probable mechanism among five of the most common mechanisms in homogeneous molecular electrochemistry. Potentially being more sensitive and capable of detecting subtle elusive features at least within our parameter range (Table S1), the established DL model can also semiquantitatively analyze competing pathways and observe the gradual transition from one mechanism to another. Additional factors that are known to impact the voltammograms, such as the 50/60 Hz noise of power-line frequency, will be incorporated into the DL model to increase its utility in practical applications. The established DL algorithm will be further refined with experimental data, being specifically targeted for algorithm development or contributed to by the general electrochemistry community via an open-access online platform. Efforts to expand the types of electrochemical mechanisms, including homogeneous, heterogeneous, stoichiometric, and catalytic transformations, will advance the utility of the algorithm. In the long run, this DL-based approach will aid if not replace extensive manual mechanistic inspections in electrochemistry. We propose that such automatic analysis will find its advantages in analyzing complex reaction schemes that may be beyond the capacity of manual analysis, such as the square diagrams with the possibility of concerted pathways in systems of proton-coupled electron transfer.^5^ The semiquantitative output of the developed DL algorithm also offers a mathematically quantified feature, which can be the subject of Bayesian optimization that seeks to maximize electrochemical transformations with optimal experimental conditions. In conjunction with automatic robotic experimentation,^17,18^ an autonomous high-throughput electrochemistry research will become feasible, where Bayesian optimization strives to maximize certain transformations with intelligently varied experimental conditions (e.g., reactant type and concentrations) in an iterative fashion by “learning” the parameter space via our DL algorithm and deciphering the partition of various reaction pathways measured in cyclic voltammetry.

## Methods

### Finite-Element Simulation of Cyclic Voltammograms

Finite-element simulations of cyclic voltammograms were conducted using COMSOL Multiphysics v5.5. The modules of Electrochemistry and Chemical Reaction Engineering were used for a one-dimensional model under the supporting electrolyte assumption with a time-dependent solver specialized for cyclic voltammetry, using an adaptive mesh with a maximal mesh size of 41 μm and a growth rate of 1.3. COMSOL simulations were iterated using COMSOL LiveLink, which implements MATLAB R2020b. Random samples of variables were realized by Python 3 scripts and fed to COMSOL via MATLAB for the simulations of at least five consecutive cycles in cyclic voltammetry. Additional sanitization was implemented after COMSOL simulation to ensure the simulated cyclic voltammograms not only satisfy the corresponding mechanism but also are electrochemically accessible. A total of about 15 000 valid simulated cases, each containing cyclic voltammograms up to six different *v* values, were conducted. The detailed model information and the constraints of variables for each specific mechanism type are provided in the Supporting Information.

### Establishment of Machine-Learning Algorithm

Simulated and experimental data were sanitized and translated into the two-dimensional matrix {*v*, *i*(*E*)}_*n*_ as reported in the main text (Figure 2A) before the implementation of machine learning. For each data point that is comprised of either simulated or experimental cyclic voltammograms at *n* number of *v* values ({*v*, *i*(*E*)}_*n*_), the current densities *i* in voltammograms were normalized as *i*_normalized_ against the largest *i* among all voltammograms in {*v*, *i*(*E*)}_*n*_, with *i*_normalized_ in the forward scan designated as a positive value. The electrochemical potentials *E* were adjusted so that the adjusted electrochemical potential *E*_adjusted_ = 0 V roughly corresponds to the potential of the studied redox couple. For voltammograms in which irreversibility precludes an accurate determination of redox potential, a rough estimate is automatically conducted based on the largest slope of the first rising redox peak. Interpolation and/or imputation of the *i*–*E* characteristics were conducted so that the two-dimensional matrix as input of the machine-learning model (Figure 2A) does not explicitly contain the information of *E*. Therefore, the use of *E*_adjusted_ in this work is mostly for presentation purposes because the absolute values of *E*_adjusted_ are not inputs of the machine-learning model and hence are not directly relevant to the automatic mechanistic analysis.

Machine-learning code was implemented on Jupyter notebooks using Python3 code. PyTorch machine-learning frameworks were used to implement the various neural networks discussed in this work. The DL algorithms are trained to take either the first or second cycles of voltammograms for the same electrochemical system at various numbers of different scan rates ({*v*, *i*(*E*, σ)}_*n*_, *n* = 1–6, σ = 0.0–1.0) and yields the vector ***y*** = {*y*_1_, *y*_2_, *y*_3_, *y*_4_, *y*_5_}. Because different starting potentials of voltammograms create additional variations for the first cycle of the voltammograms in both simulated and experimental scenarios, algorithms trained by the second cycles of voltammograms are used for the results reported here. Data of normalized cyclic voltammograms were processed by python API OpenCV to a three-dimensional tensor/matrix with a size of {6 × 3 × 500} and labels {*n,y,m*}. Here, *n* has a dimension of six correlating to the number of simulated scan rates, *m* has a dimension of 500 correlating to the 500 potential values used during resizing, and *y* has a dimension of three corresponding to *i*_for_, *i*_rev_, and *v*_*n*_, which are the forward (*i*_for_) and reverse (*i*_rev_) normalized current values for scan rate *v*_*n*_ at potential *m*. When training models with *n* < 6, the size of the tensor remains the same and empty regions are filled with zeros so that the tensor size remained {6 × 3 × 500} for all models. The input tensor was evaluated using a kernel/filter of size {6 × 3 × 7}. The kernel only views the data present in the tensor, and the filter is not changed with different values of *n*. In terms of the hyperparameters of the ResNet-18 model, the standard learning rate of 1 × 10^–3^ and standard weight decay of 1 × 10^–5^ were used for all training. It takes approximately 1 h to train the ResNet model and just seconds to predict the class of one sample. Training was performed on a machine using an Intel Xeon Silver 4214 CPU with 126 GB of RAM and an Nvidia GeForce RTX 2080 Ti GPU. A terminal training data accuracy of 99.95% during the establishment of our ResNet-18 model was achieved, and is in line with the reported test data accuracy of 98.5% (Figure 2C). Such an alignment of accuracies during model training and testing is an indication of a model that generalizes well and is not overfit. Graphs were generated using the MatPlotLib library and the PyPlot module. Training data were input with stochastically added noise after the raw/prenoise data were normalized to have a global absolute current of 1, increasing the robustness of the model to the noise encountered in real experimental data.

Final classifications were dictated by eight trained ResNet-18 models voting on a final classification to decrease the effect of randomness in individual model training. For each simulated or experimental set of cyclic voltammograms, eight individually trained ResNet-18 models will provide their ***y*** values. The values of yielded ***y*** vectors are subsequently averaged, and the maximal component in the ***y*** vector is chosen as the final predicted type of electrochemical mechanism. Relatively small yet nonzero variability of predictions exists among eight ResNet models. Statistically, the standard deviations of the yielded ***y*** vectors for a specific mechanism type are: 2.3 × 10^–3^ (*CE*), 9.3 × 10^–3^ (*EC*), 7.6 × 10^–2^ (*E*), 1.3 × 10^–4^ (*ECE*), and 3.5 × 10^–5^ (*DISP*) (*n* = 100 for each mechanism class).

### Experiments of Electrochemical Characterization

Tetraphenylporphyrin cobalt(II) (Co^II^(TPP)) (80%), tetra-*n*-butylammonium hexafluorophosphate (*n*-Bu_4_NPF_6_) (98%), and tetra-*n*-butylammonium perchlorate (*n*-Bu_4_NClO_4_) (98%) were purchased from TCI America; anhydrous diethyl ether was purchased from Fisher Scientific; ferrocenium (Fc^+^) hexafluorophosphate (98%) was purchased from Santa-Cruz Biotechnology; 1-bromobutane (*n*-BuBr) (99%), anhydrous *N*,*N*-dimethylformamide (DMF), anhydrous benzene, anhydrous acetonitrile, anhydrous tetrahydrofuran (THF), anhydrous dichloromethane, anhydrous pentane, dimanganese(0) decacarbonyl (98%), ethylenebis(diphenylphosphine) (99%), boric acid (99.5%), potassium chloride (99%), sodium hydroxide (99%), and 4-*tert*-butylcatechol (97%, HPLC) were purchased from Sigma-Aldrich. All of the chemicals were used as received unless otherwise specified below. Bu_4_NPF_6_ and Bu_4_NClO_4_ salts were recrystallized from ethanol before use. Co^II^(TPP) was recrystallized from methylene chloride before use. *n*-BuBr was fractionally distilled over CaSO_4_ under N_2_ at atmospheric pressure. The second fraction was collected at 102 °C and was dried over molecular sieves before use. THF was dried over molecular sieves before use. 4-*Tert*-butylcatechol was distilled under reduced pressure and was allowed to recrystallize under vacuum at room temperature as a white crystalline solid before use.

The Mn complex [*trans*-Mn(CO)_2_(DPPE)_2_]PF_6_ was synthesized according to a published procedure with some modifications.^32^ Dimanganese(0) decacarbonyl (0.2 g, 0.5 mmol) and DPPE (DPPE = ethylenebis(diphenylphosphine), 0.4 g, 1 mmol) were dissolved in 10 mL of benzene, and the solution was refluxed under N_2_ for 4 h. The [*trans*-Mn(CO)_2_(DPPE)][Mn(CO)_5_] salt was formed and collected as a yellowish solid. A portion of this solid (0.11 g, 0.1 mmol) was dissolved in 3 mL acetonitrile, and 1 equiv ferrocenium hexafluorophosphate (0.033 g, 0.1 mmol) was added to this solution, and the reaction mixture was stirred vigorously for 30 min. Layering diethyl ether over this reaction mixture afforded an orange-yellow solid, which upon further recrystallization with dichloromethane/pentane afforded an orange-yellow crystalline solid (0.06 g, 59%). ^31^P NMR (CDCl_3_): δ 77.9 ppm (s) and −144.3 ppm (m).

Experiments of cyclic voltammetry were performed at room temperature using a CH Instruments 630D potentiostat. Solutions in organic solvents were performed under an Ar atmosphere in a glovebox (Vigor SG1200/750TS), while aqueous experiments were performed under a N_2_ atmosphere. *iR* corrections were conducted with positive feedback compensations for the ohmic drop. Ag/Ag^+^ pseudo-reference electrode was calibrated against Fc^+^/Fc redox after electrochemical measurements.

## Supporting Information Available

Parameter ranges for the simulated voltammograms (Table S1); additional analysis of the established model (Figures S1–S3); detailed information regarding the numerical simulation of voltammograms; definitions of individual mechanisms; and procedures of randomly sampling the parameter space of individual mechanisms in cyclic voltammetry (PDF)

## Notes

B.B., W.Z., Q.G., and C.L. have filed a provisional patent.
