# Supplementary material for: Electrochemical Mechanistic Analysis from Cyclic Voltammograms Based on Deep Learning
Source: ACS Meas Sci Au. 2022 Aug 31;2(6):595–604. doi: 10.1021/acsmeasuresciau.2c00045 (PMC9783079; doi:10.1021/acsmeasuresciau.2c00045)
Supplement: Supplementary file 1 — tg2c00045_si_001.pdf [file tg2c00045_si_001.pdf]

Supplementary Information

Electrochemical mechanistic analysis from cyclic voltammograms based on deep learning

Benjamin B. Hoar,[1] Weitong Zhang,[2] Shuangning Xu,[1] Rana Deeba,[3] Cyrille Costentin,[3,4,*] Quanquan Gu,[2,*] Chong Liu[1,5,*]

[1] Department of Chemistry and Biochemistry, University of California Los Angeles, Los Angeles, California 90095, United States.

[2] Department of Computer Science, University of California Los Angeles, Los Angeles, California 90095, United States.

[3] Université Grenoble-Alpes, DCM, CNRS, 38000 Grenoble, France.

[4] Université Paris Cité, 75013 Paris, France

[5] California NanoSystems Institute, University of California Los Angeles, Los Angeles, California 90095, United States.

[*] Correspondence to: cyrille.costentin@univ-grenoble-alpes,fr (C.C.) qgu@cs.ucla.edu (Q.G.); chongliu@chem.ucla.edu (C.L.)

Index	*Page*
General considerations for the model of cyclic voltammetry	*S3*
E mechanism	*S4*
EC mechanism	*S6*
CE mechanism	*S7*
ECE mechanism	*S7*
DISP1 mechanism	*S9*
Additional considerations when sampling scan rate *v*	*S10*
Additional discussion about the number of voltammograms *n* needed for mechanism determination	*S11*
Table S1. The variables and the corresponding value ranges in the numerical simulation	*S13*
Figure S1. Exemplary simulated cyclic voltammograms with different levels of Gaussian noises	*S15*
Figure S2. Training of machine-learning algorithms for cyclic voltammetry	*S16*
Figure S3. The "importance" plots of simulated cyclic voltammograms.	*S17*
Additional References	*S18*

SUPPLEMENTARY INFORMATION

General considerations for the model of cyclic voltammetry

We established a time-dependent one-dimensional model under the supporting electrolyte assumption for the COMSOL-based finite-element simulation of cyclic voltammograms.[1-3] The model numerically simulates the oxidative electrochemical systems, in which only the reduced species (*R*) are present in the solution before cyclic voltammetry. Only the oxidative electrochemical processes are needed in the training model thanks to the process of data pre-treatment and sanitization discussed in the main text. Below are the boundary and initial conditions in specific mechanistic scenarios. The ranges in variable's values and the sampling method (linearly or logarithmically) are discussed and summarized in Table S1. As shown below, the range in variable's values could be interdependent. Such interdependence and random sampling are implemented by python 3 scripts.

Partial differential equations

$$\frac{\partial C_i}{\partial t} = D_i \frac{\partial^2 C_i}{\partial x^2} + f_i \tag{1}$$

Here f_i denotes the mechanism-specific function that describes any possible *C* step in the solution. $f_i = 0$ denotes the absence of any homogenous *C* steps.

The diffusion coefficients D_i, sampled logarithmically, are assumed to be same D for all the molecular redox species in the solution. The hypothesis of constant *D* values is reasonable for two reasons: (1) The assumed reversible and quasi-reversible *E* step suggest a small reorganization energy λ and the resultant a small change of the molecular structure. (2) The value of *D* is relatively insensitive to the changes of chemical identities since the scaling relationship between *D* and molecular weight is relatively weak ($D \propto MW^{-\frac{1}{3}}$) based on the Stokes-Einstein relationship.[4]

The initial concentration of the reduced species $C_{R,i}$ is linearly sampled from 0.1 mM to 100 mM with additional constraints listed below.

Boundary and initial conditions

Diffusion layer assumption[1,3] was implemented in the simulation. A finite diffusion layer *L* is implemented so that $x = 0$ denotes the electrode and $x = L$ denotes the boundary diffusion layer.

In Nicholson's formalism of cyclic voltammetry[5] and presented below for single-electron transfer from O to R with the period of triangular voltage wave as λ, function $S_\lambda(t)$ describes the temporal concentration variation of O in the presence of diffusion for each period of triangular voltage wave,

$$D_O \frac{\partial[O]}{\partial x} = k_s \left(\frac{C_{O,i}}{C_{R,i}}\right)^{-\alpha} [S_\lambda(t)]^{-\alpha} \left[[O]_{x=0} - \frac{C_{O,i}}{C_{R,i}} S_\lambda(t)[R]_{x=0}\right]$$

$$S_\lambda(t) = \begin{cases} e^{-at}, t < \lambda \\ e^{at-2a\lambda}, t > \lambda \end{cases}, a = \frac{Fv}{RT}$$

in which k_s is the standard rate constant of interfacial charge transfer and $\alpha = 0.5$ is the transfer coefficient.

The above expression suggests that the characteristic time constant of diffusional behavior is $\frac{RT}{Fv}$ for $S_\lambda(t)$. Therefore, in our simulation, the thickness of the diffusion layer L is adaptively chosen so that the L is more than six times of the characteristic length scale of diffusion within the noted characteristic time constant when T = 298.15 K (same below).

$$L = 6\sqrt{D \cdot \frac{RT}{Fv}} \tag{2}$$

Here the scan rate v is evenly sampled both logarithmically and linearly between 0.01 to 2 V/s. Additional algorithms to sample n number of different v values in the same simulated electrochemical systems is extensively discussed below.

In addition to the Faradaic processes simulated below, capacitive double-layer charging events are also simulated in all mechanistic scenarios, with double-layer capacitance C_{dl} randomly sampled linearly between 5 to 35 μF/cm^2 based on literature values.[6] The capcacitive current i_{dl} is simulated based the following equation,

$$i_{dl} = C_{dl} v \tag{3}$$

The current model does not include uncompensated resistance hence the *iR* drop. We contend that any serious mechanistic electrohcemical analysis should all be based on experimental data whose *iR* drop has been much minimized, if not completely mitigated, through judicious instrument setting during the experimental characterization. Moreover, as the reported deep-learning (DL)

algorithm does not intend to evaluate the reversibility of charge transfer within the *E* mechanism, the possible convolution between *iR* drop and quasi-reversible charge transfer will not negatively impact the practical utility of DL algorithm. Nonetheless, future versions of DL algorithms will consider including the impact of *iR* drops in the training data.

_E_ mechanism

$E\colon\ R \overset{E}{\leftrightarrow} O + e^{-}$

$f_R = 0,\ f_O = 0$ in eq. (1).

$[R]_{t=0} = C_{R,i}, [O]_{t=0} = 0$

The thermodynamic potential of the *O/R* redox couple is $E_{O/R} = 0$ V versus an arbitrary reference electrode. The cyclic voltammograms are simulated with a potential window in which the anodic bound $E_{window,a}$ is linearly sampled between 0.5 and 1 V vs. NHE and the cathodic bound $E_{window,c}$ is linearly sampled between −0.5 and −1 V vs. NHE. The starting potential of the cyclic voltammogram E_{start} is linearly sampled between −0.2 V vs. NHE and $E_{window,c}$. Such an arrangement of E_{start} ensures that there is minimal transient current at the beginning of voltage sweep.

Concentration-dependent Butler-Volmer equation[1] is employed to define the *E* step at the electrode interface.

$$i(t) = i_0 \left\{ \frac{[R]_{x=0}}{0.5 \cdot C_{R,i}} \exp\left[\frac{\alpha F}{RT}\left(E - E_{O/R}\right)\right] - \frac{[O]_{x=0}}{0.5 \cdot C_{R,i}} \exp\left[-\frac{(1-\alpha)F}{RT}\left(E - E_{O/R}\right)\right]\right\} \quad (4)$$

Here, $\alpha = 1/2$ and $0.5 \cdot C_{R,i}$ denotes the equilibrium concentration when $E = E_{O/R}$ and $C_R = C_O$. The exchange current density i_0 is logarithmically sampled with the upper-bound $i_{o,upper}$ and lower-bound $i_{o,lower}$.

The transfer coefficient α here is assumed to be a constant value (0.5) for 3 reasons: (1) Most of the elemental charge transfer E step in practical application possess a value of α quite close to 0.5.[1,2] Indeed, many of the classical analysis in electrochemical mechanism, such as the evaluation of Tafel slope in electrocatalysis,[1,2] can only be practically usable by assuming α = 0.5. (2) Since

the transfer coefficient α only alters the shape the waveform,[1,5] we conjecture that the impact of α could be limited in the context of differentiating *E*, *EC*, *CE*, *ECE* and *DISP* mechanisms, whose waveforms will possess much more significant variations. (3) For the semi-quantitative analysis based on our DL model as shown in Fig. 4, the relative values of **y** vectors yielded from DL algorithms remain comparable within the same electrochemical system, because the values of α in the individual *E* step remain the same under different experimental conditions. Nonetheless, in an updated version of our DL algorithm, α will be considered a variable in the future.

Following the Nicholson's formalism in cyclic voltammetry,[5] i_0 is dependent on the standard rate constant of surface change transfer k_s:

$$i_0 = k_s \cdot 0.5FC_{R,i} = \psi\sqrt{\frac{\pi F v D}{RT}} \cdot 0.5FC_{R,i} \tag{5}$$

We chose $\psi \in [10, 0.3]$ following the Nicholson's formalism[5], which corresponding to a peak separation ΔE_p = 62 ~ 120 mV in the cyclic voltammograms. We note that the upper bound of ψ values is high enough that the resultant scenarios resemble the Nernstian scenario in cyclic voltammetry in which the interfacial charge transfer is fast enough to ensure a Nernstian equilibrium for the redox species in the immediate proximity near the electrode.[1]

$$i_{0,upper} = 10 \cdot 0.5FC_{R,i}\sqrt{\frac{\pi F v D}{RT}} \quad (6a) \qquad i_{0,lower} = 0.3 \cdot 0.5FC_{R,i}\sqrt{\frac{\pi F v D}{RT}} \quad (6b)$$

As we want to ensure detectable peaks in cyclic voltammograms, additional constraint about the minimal concentration of redox *O*/*R* ($C_{R,i}$) are needed. We estimated the current densities of the redox peaks based on Randle-Sevcik equation[1,7] and ensure that the estimated current densities are approximately 5 times or more of the background current density from the capacitive double-layer charging/discharging.[1,8]

$$C_{R,i,min} = \max\left\{0.1\ mM, \frac{5}{0.446F} C_{dl} v \sqrt{\frac{RT}{FvD}}\right\} \tag{7}$$

EC mechanism

$E\colon\ R \overset{E}{\leftrightarrow} O + e^-$

$C\colon\ O \overset{C}{\leftrightarrow} A$

$f_R = 0,\ f_O = k_b[A] - k_f[O],\ f_A = k_f[O] - k_b[A]$ in eq. (1).

$$K_{O/A} \equiv \frac{k_f}{k_b}$$

$[R]_{t=0} = C_{R,i},\ [O]_{t=0} = 0, [A]_{t=0} = 0.$

Most of the constraints in the *EC* mechanism are the same as the E_r mechanism with the following additional constraints.

The equilibrium constant of the *C* step $K_{O/A}$ is logarithmically sampled between $10^{0.5} \sim 10^3$.

The kinetic rate constant of *C* step in the forward direction k_f is logarithmically sampled within the following upper and lower bound so that $\log_{10}\lambda \equiv log_{10}\left(\frac{RT}{F}\frac{k_f+k_b}{v}\right) \in [-2, 4.5]$.

$$k_{f,upper} = 10^{4.5} \cdot \frac{F}{RT} \cdot \frac{vK_{O/A}}{K_{O/A}+1} \quad (8a) \qquad k_{f,lower} = 10^{-2} \cdot \frac{F}{RT} \cdot \frac{vK_{O/A}}{K_{O/A}+1} \quad (8b)$$

The above ranges of $K_{O/A}$ and k_f values captures all of the possible variations in the *EC* mechanism as illustrated in Fig. 2.1 of Savéant's textbook (DO, KO, KG, KP, KE, and DE zones),[2] before the small value of $K_{O/A}$ leads to situations that are indeed the E_r mechanism and presented at the very upper part of that figure.

Because of the resultant potential shifts of redox peaks in the *E*C mechanism, the E_{start} is now linearly sampled between $E_{window,c}$ and $-0.2 - 0.059log_{10}K_{O/A}$ V vs. NHE.

CE mechanism

$C\colon\ A \overset{C}{\leftrightarrow} R$

$E\colon\ R \overset{E}{\leftrightarrow} O + e^-$

$f_A = k_b[R] - k_f[A],\ f_R = k_f[A] - k_b[R], f_O = 0$ in eq. (1).

$$K_{R/A} \equiv \frac{k_f}{k_b}$$

$$[A]_{t=0} = C_{R,i}\frac{1}{K_{R/A}+1},\ [R]_{t=0} = C_{R,i}\frac{K_{R/A}}{K_{R/A}+1}, [O]_{t=0} = 0.$$

Most of the constraints in the *CE* mechanism are the same as the E_r mechanism with the following additional constraints.

The equilibrium constant of the *C* step $K_{R/A}$ is logarithmically sampled between $10^{-3} \sim 10^{-0.5}$.

The kinetic rate constant of *C* step in the forward direction k_f is logarithmically sampled within the following upper and lower bound so that $\log_{10}\lambda \equiv log_{10}\left(\frac{RT}{F}\frac{k_f+k_b}{v}\right) \in [-2, 4.5]$.

$$k_{f,upper} = 10^{4.5}\cdot\frac{F}{RT}\cdot\frac{vK_{R/A}}{K_{R/A}+1} \quad (9a) \qquad k_{f,lower} = 10^{-2}\cdot\frac{F}{RT}\cdot\frac{vK_{R/A}}{K_{R/A}+1} \quad (9b)$$

The above ranges of $K_{R/A}$ and k_f values captures all of the possible variations in the *CE* mechanism as illustrated in Fig. 2.8 of Savéant's textbook (DO, KO, KG, KP, KE, and DE zones),[2] before the large value of $K_{R/A}$ leads to situations that are indeed the *E* mechanism and presented at the very upper part of that figure.

Because of the resultant potential shifts of redox peaks in the *CE* mechanism, the anodic bound of electrochemical window $E_{window,a}$ is now linearly sampled between $0.5 + 0.059log_{10}K_{R/A}$ and $1.0 + 0.059log_{10}K_{R/A}$ V vs. NHE, and the E_{start} is now linearly sampled between $E_{window,c}$ and $-0.2 + 0.059log_{10}K_{R/A}$ V vs. NHE.

ECE mechanism

E: $R1 \overset{E}{\leftrightarrow} O1 + e^-$

C: $O1 \overset{C}{\rightarrow} R2$

E: $R2 \overset{E}{\leftrightarrow} O2 + e^-$

$f_{R1} = 0,\ f_{O1} = -k[O1],\ f_{R2} = k[O1], f_{O2} = 0$ in eq. (1).

$[R1]_{t=0} = C_{R,i},\ [O1]_{t=0} = 0, [R2]_{t=0} = 0, [O2]_{t=0} = 0.$

The *E* step between *R1* and *O1* follows the same definition of *R* and *O* in the *E* mechanism.

The kinetic rate constant *k* of the *C* step is logarithmically sampled with the following constraints so that $\log_{10}\lambda \equiv log_{10}\left(\frac{RT}{F}\frac{k}{v}\right) \in [-1, 3]$

$$k_{upper} = 10^{3}\frac{F}{RT}v \qquad (10a) \qquad\qquad k_{lower} = 10^{-1}\frac{F}{RT}v \qquad (10b)$$

The selection of above *k* range covers almost all of the possible variations in the *ECE* mechanism as illustrated in Fig. 2.9 and Fig. 10a of Savéant's textbook.[2]

The *E* step between *R2* and *O2* are defined with its thermodynamic redox potential $E_{O2/R2}$ linearly sampled between −0.7 and −0.18 V vs. NHE. The electrochemical kinetics of the E_r step is defined as a concentration dependent Butler-Volmer process illustrated in eq. (4). The standard rate constant of surface change transfer $k_{s,O2/R2}$ and the corresponding exchange current density $i_{0,O2/R2}$ is defined and sampled similarly as the i_0 in the E_r mechanism.

$$i_{0,O2/R2} = k_{s,O2/R2} \cdot 0.5FC_{R,i} = \psi'\sqrt{\frac{\pi FvD}{RT}} \cdot 0.5FC_{R,i} \qquad (11)$$

When ψ' was chose as $\psi' \in [10, 0.3]$, we have,

$$i_{0,O2/R2,upper} = 10 \cdot 0.5FC_{R,i}\sqrt{\frac{\pi FvD}{RT}} \quad (12a) \qquad i_{0,O2/R2,lower} = 0.3 \cdot 0.5FC_{R,i}\sqrt{\frac{\pi FvD}{RT}} \quad (12b)$$

In order to accommodate the additional redox features, the E_{start} is now linearly sampled between $E_{window,c}$ and −0.6 V vs. NHE.

DISP1 **mechanism**

$$E:\ R1 \overset{E}{\leftrightarrow} O1 + e^{-}$$

$$C:\ O1 \overset{C}{\rightarrow} R2$$

$DISP$: $R2 + O1 \xrightarrow{DISP} O2 + R1$

$f_{R1} = k_{DISP}[R2][O1]$, $f_{O1} = -k[O1] - k_{DISP}[R2][O1]$, $f_{R2} = k[O1] - k_{DISP}[R2][O1]$, $f_{O2} = k_{DISP}[R2][O1]$ in eq. (1).

$[R1]_{t=0} = C_{R,i}$, $[O1]_{t=0} = 0, [R2]_{t=0} = 0, [O2]_{t=0} = 0.$

The *E* step between *R1* and *O1* follows the same definition of *R* and *O* in the *E* mechanism.

The E_{start} and the kinetic rate constant *k* of the *C* step follow the same definition of *k* in the *ECE* mechanism.

The kinetic rate constant k_{DISP} of the *DISP* step is logarithmically sampled with the following constraints.

$$k_{DISP,upper} = 10^2 \frac{k^{3/2}}{C_{R,i}} \sqrt{\frac{RT}{Fv}} \quad (13a) \qquad\qquad k_{DISP,upper} = 10^{-2} \frac{k^{3/2}}{C_{R,i}} \sqrt{\frac{RT}{Fv}} \quad (13b)$$

The above definition of k_{DISP} covers almost the full phase diagram noted by Savéant and coworkers[2,9] since the corresponding p_{ECE}^{DISP}, defined below, is within the range of [10^{-2}, 10^{2}].

$$p_{ECE}^{DISP} = \frac{k_{DISP}}{k^{3/2}} C_{R,i} \sqrt{\frac{Fv}{RT}} \quad (14)$$

The above definition indeed may also include scenarios that is similar, but not quite the same, to the *DISP2* mechanism,[9] when the *DISP* step is slow and rate-limiting (yet the limiting case of *DISP2* mechanism requires a reversible pre-equilibrium for the *C* step between *O1* and *R2*). Such slight ambiguity of simulated voltammograms in the training data will be addressed in future versions of the algorithm.

Additional considerations when sampling scan rate *v*

In the sampling of simulated cyclic voltammograms, variables intrinsic to the chemistry of the electrochemical systems are first sampled either linearly or logarithmically. Variables related to the electrochemical testing conditions, including E_{start}, $E_{\text{window,a}}$, $E_{\text{window,c}}$, and *v* are sampled

subsequently. Particular attention was paid the sampling of v since multiple chemistry-intrinsic variables are also dependent on the v values as shown in Table S1. Because we aim to obtain up to 6 simulated cyclic voltammograms with different v values (n = 6), an iterative process of variable samplings was implemented in the python 3 scripts as shown below.

Step 1: After the initial generation of random combinations of chemistry-related variables listed in Table S1, a medium scan rate v_{medium} is linearly sampled between 0.1 to 0.5 V/s, the range of v mostly commonly used in cyclic voltammetry. We note that as shown below v_{medium} serves as a temporal variable in the selection of v values and is not numerically used in simulation. The current densities of the redox peak is roughly estimated based on the Randle-Sevcik equation.[1,6]

$$i_{medium} = 0.446C_{R,i}\sqrt{\frac{FDv_{medium}}{RT}} \qquad (15)$$

Step 2. The maximal and minimal scan rate v_{max} and v_{min} were randomly selected based on the following constraints.

$$v_{max} = min\left\{2, \frac{RT}{\pi FD}\left(\frac{10i_0}{FC_{R,i}}\right)^2, \frac{FD}{RT}\left(\frac{0.446FC_{R,i}}{5C_{dl}}\right)^2, 10^{0.6}\cdot i_{medium}\right\} \qquad (16a)$$

$$v_{min} = max\left\{0.01, \frac{RT}{\pi FD}\left(\frac{i_0}{10FC_{R,i}}\right)^2, 10^{-0.6}\cdot i_{medium}\right\} \qquad (16b)$$

The above constraints ensure that v_{max} and v_{min} are within the ranges of 0.01 to 2 V/s, the peak separations in the Nicholson's formalism[5] will not deviate too much from the targeted values separation (ΔE_p = 62 ~ 120 mV), the voltammograms at maximal scan rates won't lead to indistinguishable redox peaks due to capacitive double-layer charging/discharging, and there is significant differences, $10^{0.6}$ ~ 4 fold difference in current densities, among the n number of simulated cyclic voltammograms.

Step 3. If $v_{max} - v_{min} \leq 0.5\ V/s$, go back to Step 1 again. Otherwise, proceed to Step 4.

Step 4. 4 more additional v values are linearly or logarithmically sampled between the v_{max} and v_{min}, leading to 6 values of v in total (n = 6).

Additional discussion about the number of voltammograms *n* needed for mechanism determination

As discussed in the main text and presented in Fig. 2e, when $n \geq 2$ the prediction accuracies of DL models trained by $\{v, i(E, \sigma)\}_n$ (n = 1 to 6, σ = 0.3) more or less remain equally satisfactory (> 95%). Such results suggest that within the tested set of simulated voltammograms, statistically on average there is diminishing returns of prediction accuracy when $n > 2$. Here we provide additional discussion and illustrate the parameter range when such a statement is applicable, given the defined parameter space of simulated voltammograms provided in the Supplementary Information and listed in Table S1.

In section "Additional considerations when sampling scan rate *v*" of the Supplementary Information, we reported the procedures of selecting the maximal and minimal values of *v* in the training set of simulated voltammograms. When $n = 2$, we propose that there exists the following approximate relationship between the two *v* values (v_{high} and v_{low}) and the medium value v_{medium}:

$$v_{medium} \approx \frac{v_{high} + v_{low}}{2} \in [0.1, 0.5]\ V/s \quad (17)$$

Here the "≈" sign suggests that the above relationship is a statistically approximation given that v_{high} and v_{low} are randomly sampled around the value of v_{medium}.

Hence, the approximate range of v_{high} and v_{low} can be defined as:

$$v_{high} = min\left\{2, \frac{RT}{\pi FD}\left(\frac{10i_0}{FC_{R,i}}\right)^2, \frac{FD}{RT}\left(\frac{0.446FC_{R,i}}{5C_{dl}}\right)^2, 10^{0.6} \cdot 0.446C_{R,i}\sqrt{\frac{FD(v_{high} + v_{low})}{2RT}}\right\}$$

$$v_{low} = max\left\{0.01, \frac{RT}{\pi FD}\left(\frac{i_0}{10FC_{R,i}}\right)^2, 10^{-0.6} \cdot 0.446C_{R,i}\sqrt{\frac{FD(v_{high} + v_{low})}{2RT}}\right\}$$

$$v_{max} - v_{min} \leq 0.5\ V/s \quad (18a, 18b, 18c)$$

Equation (18a), (18b), and (18c) provide an approximate empirical range of v_{high} and v_{low} values in order to satisfy the defined training data set of voltammograms and hence offer good accuracy

of mechanistic prediction based on our DL model. The above relationships indicate that v_{high} and v_{low} values are dependent on the redox species' concentration ($C_{R,i}$) and diffusion coefficient (D), the electrodes' double-layer capacitance (C_{dl}), and the exchange current density (i_0) hence the standard rate constant of interfacial charge transfer (k_s) based on equation (5). A combination of experimental parameters ($C_{R,i}$ and C_{dl}) and redox's intrinsic properties (D and k_s) determines the values of v_{high} and v_{low} for effective discernment of electrochemical mechanisms.

Table S1. The variables and the corresponding value ranges in the numerical simulation

Variable	Value ranges	Constraints
***E_r* mechanism**		
v	0.01 ~ 2 V/s [a,b]	Default setting, see specific mechanisms below if noted
$C_{R,i}$	$C_{R,i,min}$ ~ 100 mM [a]	$C_{R,i,min} = \max\left\{0.1\,mM, \frac{5}{0.446F} C_{dl} v \sqrt{\frac{RT}{FvD}}\right\}$
D	1×10^{-7} ~ 1×10^{-4} cm^2/s [a,c]	
L	N.A.	$L = 6\sqrt{D \cdot \frac{RT}{Fv}}$
C_{dl}	5 ~ 35 μF/cm^2 [b]	
$E_{window,a}$	0.5 ~ 1 V vs. NHE [b]	Default setting, see specific mechanisms below if noted
$E_{window,c}$	−0.9 ~ −1.4 V vs. NHE [b]	
E_{start}	$E_{window,c}$ ~ −0.2 V vs. NHE [b]	Default setting, see specific mechanisms below if noted
i_0	$i_{0,lower}$ ~ $i_{0,upper}$ [a]	$i_{0,lower} = 0.3 \cdot 0.5FC_{R,i}\sqrt{\frac{\pi FvD}{RT}}$<br>$i_{0,upper} = 10 \cdot 0.5FC_{R,i}\sqrt{\frac{\pi FvD}{RT}}$<br>Default setting, see specific mechanisms below if noted
***E_rC_r* mechanism**		
$K_{O/A}$	$10^{0.5}$ ~ 10^{3} [a]	
k_f	$k_{f,lower}$ ~ $k_{f,upper}$ [a]	$k_{f,lower} = 10^{-2} \cdot \frac{F}{RT} \cdot \frac{vK_{O/A}}{K_{O/A}+1}$<br>$k_{f,upper} = 10^{4.5} \cdot \frac{F}{RT} \cdot \frac{vK_{O/A}}{K_{O/A}+1}$
E_{start}	$E_{window,c}$ ~ $-0.2 - 0.059log_{10}K_{O/A}$ V vs. NHE [b]	
***C_rE_r* mechanism**		
$K_{R/A}$	10^{-3} ~ $10^{-0.5}$ [a]	
k_f	$k_{f,lower}$ ~ $k_{f,upper}$ [a]	$k_{f,lower} = 10^{-2} \cdot \frac{F}{RT} \cdot \frac{vK_{R/A}}{K_{R/A}+1}$<br>$k_{f,upper} = 10^{4.5} \cdot \frac{F}{RT} \cdot \frac{vK_{R/A}}{K_{R/A}+1}$
$E_{window,a}$	$0.5 + 0.059log_{10}K_{R/A}$ ~ $1.0 + 0.059log_{10}K_{R/A}$ vs. NHE [b]	
E_{start}	$E_{window,c}$ ~ $-0.2 + 0.059log_{10}K_{R/A}$ V vs. NHE [b]	
***ECE* mechanism**		
k	k_{lower} ~ k_{upper} [a]	$k_{upper} = 10^{3}\frac{F}{RT}v$

		$k_{lower} = 10^{-1} \frac{F}{RT} v$
$E_{O2/R2}$	−0.7 ~ −0.18 V vs. NHE [b]	
$i_{0,O2/R2}$	$i_{0,O2/R2,lower}$ ~ $i_{0,O2/R2,upper}$ [a]	$i_{0,O2/R2,upper} = 10 \cdot 0.5FC_{R,i}\sqrt{\frac{\pi FvD}{RT}}$ $i_{0,O2/R2,lower} = 0.3 \cdot 0.5FC_{R,i}\sqrt{\frac{\pi FvD}{RT}}$
E_{start}	$E_{window,c}$ ~ −0.6 vs. NHE [b]	
***DISP* mechanism**		
k	$k_{lower} \sim k_{upper}$ [a]	$k_{upper} = 10^{3} \frac{F}{RT} v$ $k_{lower} = 10^{-1} \frac{F}{RT} v$
E_{start}	$E_{window,c}$ ~ −0.6 vs. NHE [b]	
k_{DISP}	$k_{DISP,lower}$ ~ $k_{DISP,upper}$ [a]	$k_{DISP,upper} = 10^{2} \frac{k^{3/2}}{C_{R,i}}\sqrt{\frac{RT}{Fv}}$ $k_{DISP,upper} = 10^{-2} \frac{k^{3/2}}{C_{R,i}}\sqrt{\frac{RT}{Fv}}$

[a] Variable values are randomly sampled logarithmically
[b] Variable values are randomly sampled linearly.

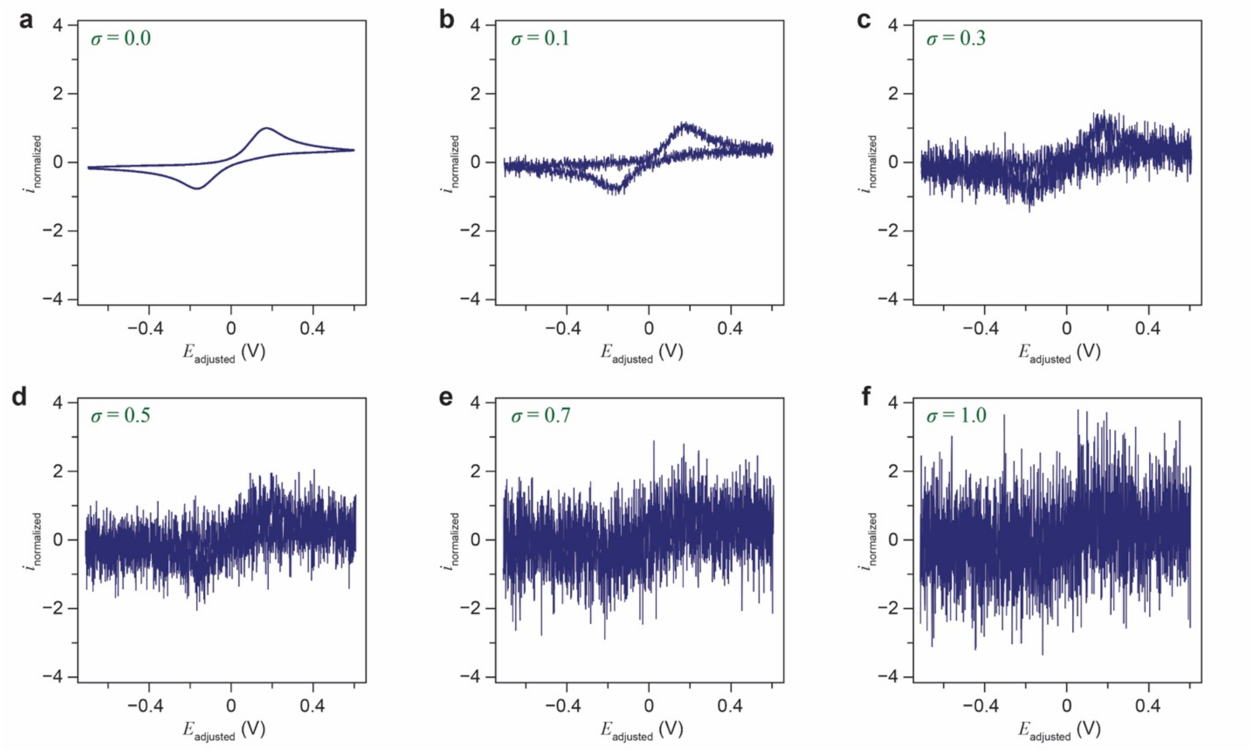

Figure S1. Exemplary simulated cyclic voltammograms with different levels of Gaussian noises. The same simulated cyclic voltammograms added with Gaussian noises whose standard deviations σ = 0.0 (**a**), 0.1 (**b**), 0.3 (**c**), 0.5 (**d**), 0.7 (**e**), and 1.0 (**f**). The voltammograms of the second cycles are displayed.

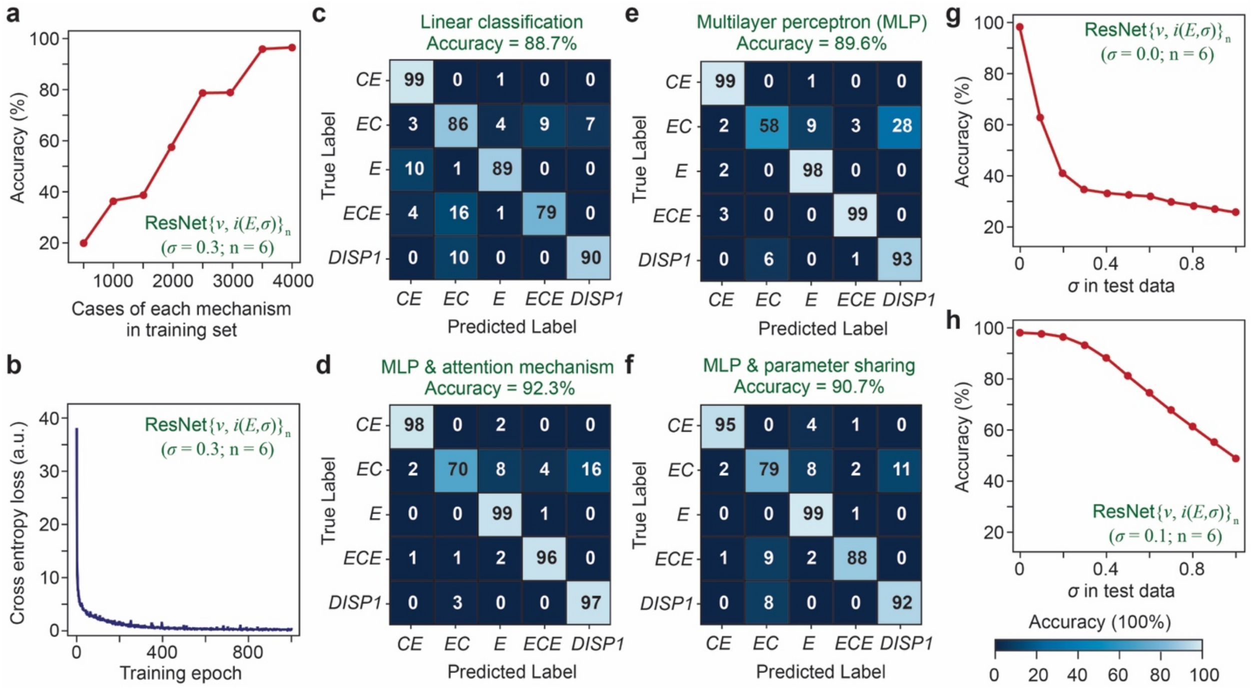

Figure S2. Training of machine-learning algorithms for cyclic voltammetry. **a**, Accuracies of RetNet{v, i(E, σ)}$_n$ (σ = 0.3; n = 6) trained by varying numbers of simulated electrochemical systems in each mechanism in the training set. **b**, Cross entropy loss, a surrogate of the algorithms' accuracy in the training process, as a function of training epochs for ResNet{v, i(E, σ)}$_n$ (σ = 0.3; n = 6). **c** to **f**, the confusion matrices and the overall accuracy of various machine-learning models trained by simulated cyclic voltammograms. Training data, {v, i(E, σ)}$_n$ (σ = 0.3; n = 6). **g** and **h**, the accuracies of the DL models trained by {v, i(E, σ)}$_n$ (σ = 0.0; n = 6) and v, i(E, σ)}$_n$ (σ = 0.1; n = 6), respectively, when tested with simulated voltammograms with varying values of n and σ. Here the "linear classification" model is the simplest as it require that the high dimensional data be linearly separable. In the fully-connected MLP every node of every layer is connected to every node of the layers before it and after it. Instead, the ResNets architecture are not fully connected and further have residual layers in place which act as mechanisms to prevent training problems such as exploding and vanishing gradients that can occur during the learning process/optimization of network architectures.

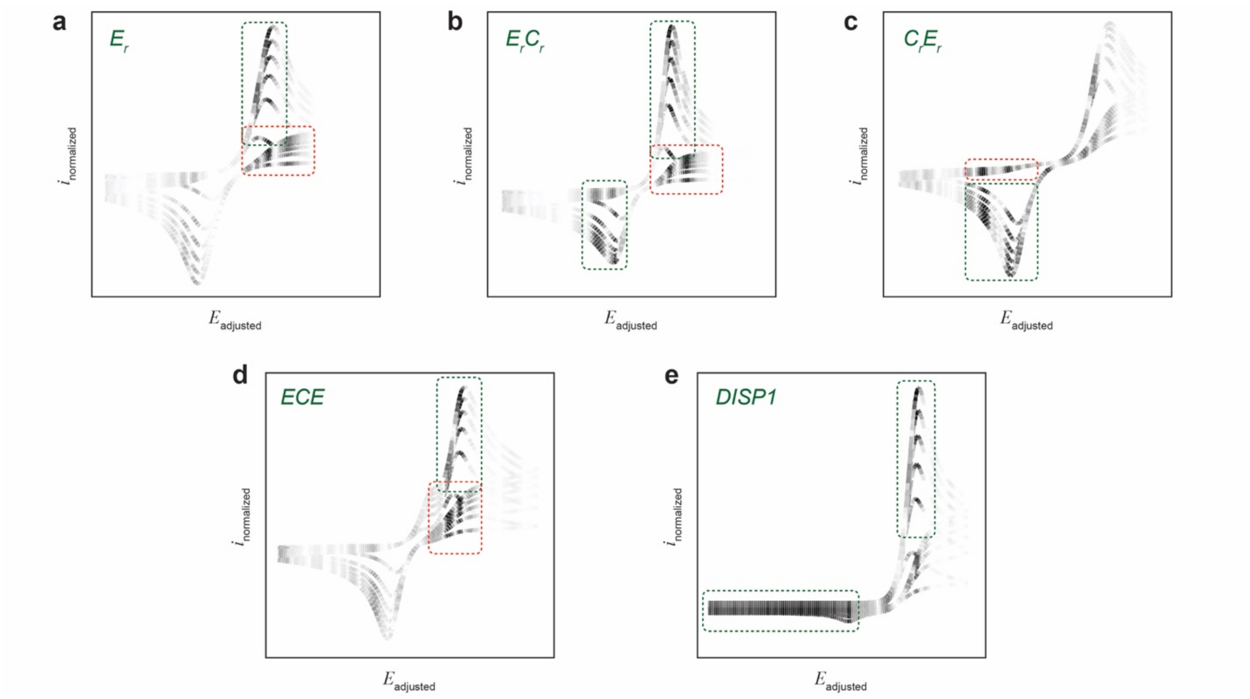

Figure S3. The “importance” plots of simulated cyclic voltammograms. Exemplary simulated cyclic voltammograms (σ = 0.0) and the corresponding “importance” in RetNet$\{v, i(E, \sigma)\}_n$ (σ = 0.3; n = 6) for *E* (**a**), *EC* (**b**), *CE* (**c**), *ECE* (**d**), and *DISP1* (**e**) mechanisms. The “importance” towards the DL model in expected (green) and somewhat unexpected (red) parts in the voltammograms are highlighted. The voltammograms of the second cycles are displayed and analyzed.

Additional References

1 Bard, A. J. & Faulkner, L. R. *Electrochemical Methods: Fundamentals and Applications*. 2 edn, (John Wiley & Sons, inc., 2001).

2 Savéant, J.-M. & Costentin, C. *Elements of Molecular and Biomolecular Electrochemistry: An Electrochemical Approach to Electron Transfer Chemistry*. 2nd edn, (John Wiley & Sons, Inc., 2019).

3 Newman, J. & Thomas-Alyea, K. E. *Electrochemical Systems*. 3rd edn, (Wiley, 2004).

4 Young, M. E., Carroad, P. A. & Bell, R. L. Estimation of diffusion coefficients of proteins. *Biotechnol Bioeng* **22**, 947−955 (1980).

5 Nicholson, R. S. Theory and Application of Cyclic Voltammetry for Measurement of Electrode Reaction Kinetics. *Anal Chem* **37**, 1351−1355 (1965).

6 Yoon, Y., Yan, B. & Surendranath, Y. Suppressing Ion Transfer Enables Versatile Measurements of Electrochemical Surface Area for Intrinsic Activity Comparisons. *J Am Chem Soc* **140**, 2397−2400 (2018).

7 Elgrishi, N. *et al.* A Practical Beginner's Guide to Cyclic Voltammetry. *J Chem Educ* **95**, 197−206 (2018).

8 Gao, R., Edwards, M. A., Harris, J. M. & White, H. S. Shot noise sets the limit of quantification in electrochemical measurements. *Curr Opin Electrochem* **22**, 170−177 (2020).

9 Amatore, C., Gareil, M. & Savéant, J. M. Homogeneous vs. heterogeneous electron transfer in electrochemical reactions: Application to the electrohydrogenation of anthracene and related reactions. *J. Electroanal Chem Interf Electrochem* **147**, 1−38 (1983).
